# Pairing-Dependent Plasticity in a Dissected Fly Brain Is Input-Specific and Requires Synaptic CaMKII Enrichment and Nighttime Sleep

**DOI:** 10.1523/JNEUROSCI.0144-22.2022

**Published:** 2022-05-25

**Authors:** Mohamed Adel, Nannan Chen, Yunpeng Zhang, Martha L. Reed, Christina Quasney, Leslie C. Griffith

**Affiliations:** Department of Biology and Volen National Center for Complex Systems, Brandeis University, Waltham, Massachusetts 02454-9110

**Keywords:** brain explant, CaMKII, *Drosophila* mushroom body, learning and memory circuit, neural plasticity, sleep

## Abstract

In *Drosophila*, *in vivo* functional imaging studies revealed that associative memory formation is coupled to a cascade of neural plasticity events in distinct compartments of the mushroom body (MB). In-depth investigation of the circuit dynamics, however, will require an *ex vivo* model that faithfully mirrors these events to allow direct manipulations of circuit elements that are inaccessible in the intact fly. The current *ex vivo* models have been able to reproduce the fundamental plasticity of aversive short-term memory, a potentiation of the MB intrinsic neuron (Kenyon cells [KCs]) responses after artificial learning *ex vivo*. However, this potentiation showed different localization and encoding properties from those reported *in vivo* and failed to generate the previously reported suppression plasticity in the MB output neurons (MBONs). Here, we develop an *ex vivo* model using the female *Drosophila* brain that recapitulates behaviorally evoked plasticity in the KCs and MBONs. We demonstrate that this plasticity accurately localizes to the MB α′3 compartment and is encoded by a coincidence between KC activation and dopaminergic input. The formed plasticity is input-specific, requiring pairing of the conditioned stimulus and unconditioned stimulus pathways; hence, we name it pairing-dependent plasticity. Pairing-dependent plasticity formation requires an intact *CaMKII* gene and is blocked by previous-night sleep deprivation but is rescued by rebound sleep. In conclusion, we show that our *ex vivo* preparation recapitulates behavioral and imaging results from intact animals and can provide new insights into mechanisms of memory formation at the level of molecules, circuits, and brain state.

**SIGNIFICANCE STATEMENT** The mammalian *ex vivo* LTP model enabled in-depth investigation of the hippocampal memory circuit. We develop a parallel model to study the *Drosophila* mushroom body (MB) memory circuit. Pairing activation of the conditioned stimulus and unconditioned stimulus pathways in dissected brains induces a potentiation pairing-dependent plasticity (PDP) in the axons of α′β′ Kenyon cells and a suppression PDP in the dendrites of their postsynaptic MB output neurons, localized in the MB α′3 compartment. This PDP is input-specific and requires the 3′ untranslated region of *CaMKII*. Interestingly, *ex vivo* PDP carries information about the animal's experience before dissection; brains from sleep-deprived animals fail to form PDP, whereas those from animals who recovered 2 h of their lost sleep form PDP.

## Introduction

A neutral experience (conditioned stimulus [CS]) can be remembered as positive or negative if closely followed by rewarding or punishing reinforcement (unconditioned stimulus [US]). The ability to form this type of “associative” memory is phylogenetically conserved; *Drosophila* form robust associative memories (Tully and Quinn, 1985), most of which are encoded and stored in the mushroom body (MB) (de Belle and Heisenberg, 1994). The MB is a higher brain structure made of 15 distinct compartments. Each compartment is built on a scaffold of axons of one of the three main types of Kenyon cells (KCs; αβ, α′β′, and γ). The KCs connect to MB output neurons (MBONs), which project out of the MB to bias behavior (Aso et al., 2014a,b; F. Li et al., 2020). The KC→MBON synapses are modulated by dopaminergic neurons.

During aversive olfactory associative learning, an odor (the CS) activates a sparse group of KCs, such that this odor identity is represented across all MB compartments (Turner et al., 2008; Lin et al., 2014). Simultaneously, dopaminergic neurons from the protocerebral posterior lateral (PPL1) cluster are activated by the US, encoding negative prediction errors in MB compartments (Riemensperger et al., 2005; Schroll et al., 2006; Claridge-Chang et al., 2009; Mao and Davis, 2009; Aso et al., 2012). When KC activation and the dopaminergic signal coincide within a compartment, the KC→MBON synapses in that compartment are depressed, biasing the circuit output to aversion (Sejourne et al., 2011; Cohn et al., 2015; Hige et al., 2015; Owald and Waddell, 2015; Owald et al., 2015).

Many studies have investigated the properties of this circuitry using *in vivo* calcium imaging in intact animals (for review, see Adel and Griffith, 2021). In contrast, explanted brains have been used mostly for establishing connectivity between neurons or interrogating a specific biochemical pathway; only a few studies have attempted to understand memory circuit logic *ex vivo* (Wang et al., 2008; Ueno et al., 2013, 2017; Suzuki-Sawano et al., 2017). In the best-developed paradigm, Ueno et al. (2013) observed a potentiation of KC responses in the tips of the MB vertical lobes which they termed “long-term enhancement” (LTE). This laid the groundwork for developing *ex vivo* models of this circuit, but there were major differences between LTE and associative memory observed in intact animals. The most significant were that the plasticity was not specific to the α′β′ KCs and that dopamine release by the US was not observed; it was only seen after CS+US coincidence (Ueno et al., 2013, 2017).

In this study, we establish an *ex vivo* paradigm that resolves these discrepancies and exhibits the cardinal features of associative learning. We show that pairing odor and punishment pathway activation in dissected brains results in a localized potentiation of the α′β′ KCs and suppression of their postsynaptic MBONs in the α′3 compartment. Because both KC potentiation and MBON suppression are strictly dependent on temporal coincidence of the CS and US, we term this paradigm “pairing-dependent plasticity” (PDP). We show that, like the CS specificity of associative memories, PDP is specific to the subset of odor-representing projection neurons activated during the artificial training. We also provide evidence that dopamine is released by activation of the US pathway and does not require CS+US coincidence.

This *ex vivo* paradigm can be used for obtaining new mechanistic insight into memory formation at the molecular and circuit levels. We present data indicating that the 3′UTR of the *CaMKII* gene is critical for short-term memory (STM) formation and that the primacy of α′ compartment plasticity in learning is because of differences in input/response relationships between α and α′. Finally, we demonstrate that the ability of the *ex vivo* brain to be plastic can be influenced by prior *in vivo* experience, as we report that brains of sleep-deprived flies fail to form PDP, but as little as 2 h of recovery sleep rescues this learning impairment.

## Materials and Methods

### Fly husbandry

All fly stocks were cultured on standard food at room temperature. Experimental flies were kept at 25°C and 70% relative humidity on a 12 h light, 12 h dark period. Fly lines used in this study include *VT030559-GAL4, MB027B split-GAL4* (Aso et al., 2014a), *GH146-GAL4, UAS-GCaMP6f, 20*× *UAS-GCaMP6f, UAS-jRCaMP1a, LexAop-P2X_2_. TH-lexA* was gifted to us from the Davis laboratory (Berry et al., 2015) and *UAS-GRAB^DA2m^* was gifted from the Li laboratory (Sun et al., 2020). *CaMKII^UDel^* flies are described by Chen et al. (2022).

### *Ex vivo* imaging and electrical stimulations

Brains from 4- to 8-d-old female flies were dissected in ice-cold HL3 medium (70 mm NaCl, 115 mm sucrose, 5 mm KCl, 20 mm MgCl_2_, 1.8 mm CaCl_2_, 10 mm NaHCO_3_, 5 mm trehalose, 5 mm HEPES, osmolarity: 395.4 mOsm; pH 7.3) (Stewart et al., 1994). Brains were then transferred to an imaging chamber containing fresh HL3 saline and immobilized using tungsten pins over the optic lobes. In the case of paired stimulations of the antennal lobe (AL) and the ascending fibers of the ventral nerve cord (AFV), the brain was dissected with the ventral nerve cord attached. The ventral nerve cord was then cut using sharp scissors near its base, leaving one end of the AFV free. Glass suction microelectrodes were used to apply the electrical stimulation to either the AL or the AFV. Because odor information is randomly encoded in the MB (Bhandawat et al., 2007; Ito et al., 2008; Turner et al., 2008; Honegger et al., 2011; Lei et al., 2013), we did not target specific AL glomeruli across different animals. However, the properties of the stimulation and the electrode were kept the same across all experiments unless noted otherwise. Based on the size of the AL electrode tip and the distribution of AL calcium responses to AL stimulation (see Fig. 3*B–D*), we estimate that 20%-25% of the ipsilateral AL projection neurons are activated with our AL stimulation protocol. Brains were always perfused with fresh saline throughout the experiment with a flow rate of ∼2 drops per second.

In pre-induction and post-induction AL stimulations, 5 trains of 20 pulses at 100 Hz were applied. Pulse width is 1 ms and interpulse interval is 9 ms. Intertrain interval is 15 s. Stimulation strength is 100 (low input stimulation) or 200 (high input stimulation) µAmps. During induction, 12 AL stimulation trains were applied with 5 s intertrain interval. Regarding the AL electrode size, we noticed a relationship between the diameter of the AL electrode tip and the input current such that applying a 100 µAmps input stimulation via a large electrode tip diameter has a similar effect on baseline KC calcium responses and on the resultant plasticity as applying a 200 µAmps via a small electrode tip diameter. For example, with a 100 µAmps input stimulation, PDP can be formed in the MB α3 compartment if the diameter of the AL electrode is significantly enlarged. Therefore, to minimize variability within the same dataset, all electrodes used in any experiment in this study were made at the same time using a p-97 micropipette puller (Sutter Instruments) before data collection. To minimize variability across the different experiments, we kept the AL electrode size approximately one-fourth of the AL size.

AFV stimulation was similar to AL stimulation, but the diameter of the AFV stimulation electrode was large enough to suck in the free end of the AFV. In GRAB_DA2m_ experiments, AFV stimulation strength was adjusted to be above the threshold of KC GRAB_DA2m_ responses; it varied between 500 and 1000 µAmps. We noted that AFV stimulation under these conditions did not produce a GRAB_DA2m_ response in horizontal lobes, suggesting that, while PPL1 neurons were stimulated, PAM neurons were not. Whether this reflects differences in circuitry or differences in relative excitability (we are using HL3) is unknown. In AL pairing experiments, we noted in tests of the AFV electrode that there were MB GCaMP responses in some animals, but not all (2 of 8 had no response). In no case, however, did AFV stimulation alone cause PDP, suggesting that AFV-stimulated MB calcium increases are not able to act as a CS. Plasticity has been observed to be dependent on calcium entry pathway previously in mammalian brain (Deisseroth et al., 1998).

Images were captured using an ORCA Flash4.0 V3 sCMOS camera at 200 frames per second (except in the MBON imaging experiment using *MB027B split-GAL4* > *20*×-*UAS-GCaMP6f*, frame rate was reduced to 2 frames per second) with a 40× water immersion lens on an Olympus upright microscope BX50W1. Images were collected as 512 × 512 resolution and a binning factor of 2 × 2. Imaging was done using the HCImage Live software. Excitation of the used florescent sensors was done using the CoolLED pE-4000 LED source. For GCaMP and GRAB_DA2m_, the 470 nm LED channel was used with an excitation filter Chroma 450/50 and emission filter FF01-525/45-25. jRCaMP excitation was done using the 550 nm LED channel and the excitation filter FF01-530/43-25 and an emission filter FF01-607/36-25. Calcium traces at every frame were calculated as ΔF/F_0_ = (F – F_0_)/F_0_, where F is the florescence value at a given frame and F_0_ is the florescence value at baseline. Peak response to a stimulation was calculated by subtracting the average ΔF/F_0_ during the last second before a stimulation from the peak ΔF/F_0_ during stimulation. The responses to AL stimulations before and 15 min after induction were averaged to calculate the pre- and post-responses, respectively. PDP values or ΔF/F relative change was calculated as (post – pre)/pre.

The isolated pulse stimulator model 2100 and the perfusion system ValveLink 8.2 were triggered using a custom program written and controlled by the pClamp 11 software and the Digidata1550A digitizer.

### Behavioral experiments

Aversive learning experiments were performed in an environmental room in red light at 25°C with 65% humidity. Flies (mixed males and females) were between 4- and 14-d-old. Flies were given at least 10 min acclimation period in room before training or testing. Data for each experiment were pooled from at least three independent experimental days. The learning assays were performed as described by the Quinn laboratory (Tully and Quinn, 1985). The US was provided as 12 1 s 90 or 60 V shocks during the 1 min CS-US pairing; 10% 4-methylcyclohexanol and 3-octanol OCT were used as the CS odors. Flies were then given a 2 min rest. Testing involved 2 min of simultaneous exposure to CS odors, after which flies choosing either odor were counted. A performance index was calculated for each trial as (number of flies choosing the conditioned odor) – (number of flies choosing the not-conditioned odor)/(total number of flies). This performance index was averaged between reciprocal trials where one of the odors was conditioned in one trial and the other odor was conditioned in the other to calculate the Learning Index. To confirm the *CaMKII^UDel^* flies' sensitivity to electric shock, 2 min preference tests were performed during which flies chose between a stimulus vial (24 spaced 1 s 90 V shocks) and a neutral vial.

### Sleep assay

Sleep deprivation was done in 25°C incubators on a 12 h light, 12 h dark cycles. Mated female flies were individually loaded into glass sleep tubes containing a food mixture of 5% sucrose and 2% agar. Drosophila Activity monitors system (TriKinetics) was used to measure sleep. Sleep was defined as inactivity bouts of 5 or more minutes (Hendricks et al., 2000; Shaw et al., 2000). Flies were sleep-deprived by turning on the shaker between ZT12 and ZT24. Drosophila Activity monitor data were analyzed using a custom MATLAB program (Donelson et al., 2012).

### Immunostaining

Adult fly brains were dissected in cold Schneider's Insect Medium (Sigma, S0146), and then fixed in 4% PFA solution for 30 min at room temperature. Fixed brains were washed 3 × 30 min in 0.5% Triton-PBS (PBST) solution, blocked in 10% normal goat serum solution for 1 h, and incubated in mouse anti-CaMKII antibody (1:10 000, Cosmo) for 3 d. CaMKII antibody solutions were removed, and samples washed in PBST solution for 3 × 30 min. Samples were then incubated in AlexaFluor-633 anti-mouse antibody (Invitrogen) overnight, then washed 3 × 30 min in PBST solution and mounted in the Vectashield mounting medium. Images were taken under a 20× objective lens with the same settings using a Leica SP5 confocal microscope. The images were analyzed by ImageJ software. For the intensity of MB regions, the middle slices were selected as the representative pictures, and mean intensity of all MB lobes was quantified.

### Immunoblotting

A total of 100 5-d-old *Canton-S* WT or *CaMKII^UDel^* flies (mixed males and females) were frozen on dry ice and vortexed to remove heads. Fly heads were separated from the fly bodies using a sieve. The heads were then homogenized in loading buffer (4× Bolt LDS, Invitrogen, Novex with 5% β-mercaptoethanol added) and heated for 10 min. Proteins were separated by SDS-PAGE (Bolt, Bis-Tris Protein Gels, Invitrogen) and transferred to a nitrocellulose membrane (GE Healthcare). Membrane was blocked (Blocking Buffer for Fluorescent Western Blotting, Rockland Immunochemicals) and then incubated with Anti-dCaMKII Clone 18 (1:1000, CosmoBio) and anti-actin mAb C4 (1:1000, Millipore). The secondary antibody used was DyLight 680 mouse. Membrane was imaged using ChemiDoc system from Bio-Rad. Intensity of bands was calculated using Adj. Volume (Int) within the ImageLab 6.0 software. Intensity of the CaMKII band was normalized to that of actin in the same lane.

### Experimental design and statistical analysis

Female flies were used in all imaging experiments because of their larger size. Both males and females aged 4-8 d were used in other experiments unless noted otherwise. All statistical analyses were performed in Prism 9 software. All tests were two-tailed, and confidence levels were set at α = 0.05. Normality of statistical data was determined via the Shapiro–Wilk test (α = 0.05). Parametric tests were used for all experiments, except the immunoblotting experiment where nonparametric tests were used. The statistical tests, *p* values, sample sizes, and other statistical information for each experiment are listed in the figure legends. *Post hoc* analysis information for Figures 2*G* and 5*F* is listed in Tables 1 and 2, respectively.

**Table 1.** Tukey's *post hoc* results for Figure 2*G*^*a*^

	α′3					α3				
Lobe Comparison	N1	N2	*q*	DF	*p*	N1	N2	*q*	DF	*p*
DA alone (1 μA) vs unpaired (1 μA)	4	4	1.591	68	0.9683	4	4	0.8009	68	0.9997
DA alone (1 μA) vs paired (1 μA)	4	4	1.381	68	0.9868	4	4	0.7118	68	0.9999
DA alone (1 μA) vs DA alone (5 μA)	4	4	0.5161	68	>0.9999	4	4	0.2955	68	>0.9999
DA alone (1 μA) vs unpaired (5 μA)	4	4	3.338	68	0.3226	4	4	0.937	68	0.9991
DA alone (1 μA) vs paired (5 μA)	4	6	4.831	68	0.028	4	6	1.625	68	0.9641
DA alone (1 μA) vs unpaired (10 μA)	4	6	3.055	68	0.4426	4	6	3.592	68	0.2319
DA alone (1 μA) vs paired (10 μA)	4	6	5.119	68	0.0155	4	6	1.351	68	0.9886
DA alone (1 μA) vs DA alone (10 μA)	4	5	0.4062	68	>0.9999	4	5	0.3997	68	>0.9999
Unpaired (1 μA) vs paired (1 μA)	4	4	0.2098	68	>0.9999	4	4	0.0891	68	>0.9999
Unpaired (1 μA) vs DA alone (5 μA)	4	4	2.107	68	0.8563	4	4	1.096	68	0.9972
Unpaired (1 μA) vs unpaired (5 μA)	4	4	1.746	68	0.9457	4	4	0.1361	68	>0.9999
Unpaired (1 μA) vs paired (5 μA)	4	6	6.574	68	0.0005	4	6	2.502	68	0.7017
Unpaired (1 μA) vs unpaired (10 μA)	4	6	1.312	68	0.9905	4	6	2.715	68	0.6029
Unpaired (1 μA) vs paired (10 μA)	4	6	6.862	68	0.0002	4	6	0.4732	68	>0.9999
Unpaired (1 μA) vs DA alone (10 μA)	4	5	1.271	68	0.9923	4	5	0.4445	68	>0.9999
Paired (1 μA) vs DA alone (5 μA)	4	4	1.897	68	0.9148	4	4	1.007	68	0.9985
Paired (1 μA) vs unpaired (5 μA)	4	4	1.956	68	0.9003	4	4	0.2252	68	>0.9999
Paired (1 μA) vs paired (5 μA)	4	6	6.344	68	0.0009	4	6	2.404	68	0.7443
Paired (1 μA) vs unpaired (10 μA)	4	6	1.542	68	0.9737	4	6	2.812	68	0.5564
Paired (1 μA) vs paired (10 μA)	4	6	6.632	68	0.0004	4	6	0.5708	68	>0.9999
Paired (1 μA) vs DA alone (10 μA)	4	5	1.05	68	0.9979	4	5	0.3506	68	>0.9999
DA alone (5 μA) vs unpaired (5 μA)	4	4	3.854	68	0.1587	4	4	1.232	68	0.9938
DA alone (5 μA) vs paired (5 μA)	4	6	4.265	68	0.0808	4	6	1.301	68	0.9911
DA alone (5 μA) vs unpaired (10 μA)	4	6	3.621	68	0.223	4	6	3.916	68	0.1441
DA alone (5 μA) vs paired (10 μA)	4	6	4.554	68	0.0479	4	6	1.674	68	0.9573
DA alone (5 μA) vs DA alone (10 μA)	4	5	0.9503	68	0.999	4	5	0.7112	68	0.9999
Unpaired (5 μA) vs paired (5 μA)	4	6	8.487	68	<0.0001	4	6	2.651	68	0.633
Unpaired (5 μA) vs unpaired (10 μA)	4	6	0.6008	68	>0.9999	4	6	2.566	68	0.6728
Unpaired (5 μA) vs paired (10 μA)	4	6	8.775	68	<0.0001	4	6	0.3241	68	>0.9999
Unpaired (5 μA) vs DA alone (10 μA)	4	5	3.112	68	0.4172	4	5	0.5879	68	>0.9999
Paired (5 μA) vs unpaired (10 μA)	6	6	8.817	68	<0.0001	6	6	5.833	68	0.0031
Paired (5 μA) vs paired (10 μA)	6	6	0.3224	68	>0.9999	6	6	3.326	68	0.3269
Paired (5 μA) vs DA alone (10 μA)	6	5	5.6	68	0.0054	6	5	2.175	68	0.8336
Unpaired (10 μA) vs paired (10 μA)	6	6	9.139	68	<0.0001	6	6	2.506	68	0.6998
Unpaired (10 μA) vs DA alone (10 μA)	6	5	2.807	68	0.559	6	5	3.386	68	0.3038
Paired (10 μA) vs DA alone (10 μA)	6	5	5.907	68	0.0026	6	5	0.9969	68	0.9986

^a^Statistical data for each comparison in Figure 2*G* in α′3 and α3 compartments. N1, Sample size of first group; N2, sample size of second group; *q*, value of Studentized range distribution.

**Table 2.** Sidak *post hoc* results for Figure 5*F*^*a*^

Comparison	N1	N2	*t*	DF	*p*
WT:α′3 unpaired vs CaMKIIUDel:α′3 unpaired	6	6	0.5011	40	>0.9999
WT:α′3 paired vs CaMKIIUDel:α′3 paired	6	6	3.417	40	0.0174
WT:α3 unpaired vs CaMKIIUDel:α3 unpaired	6	6	0.7521	40	0.9993
WT:α3 paired vs CaMKIIUDel:α3 paired	6	6	2.708	40	0.1127
WT:α′3 unpaired vs WT:α′3 paired	6	6	7.344	40	<0.0001
WT:α3 unpaired vs WT:α3 paired	6	6	4.116	40	0.0022
CaMKIIUDel:α′3 unpaired vs CaMKIIUDel:α′3 paired	6	6	4.428	40	0.0009
CaMKIIUDel:α3 unpaired vs CaMKIIUDel:α3 paired	6	6	0.6559	40	0.9998
WT:α′3 unpaired vs WT:α3 unpaired	6	6	0.9692	40	0.993
WT:α′3 paired vs WT:α3 paired	6	6	4.198	40	0.0017
CaMKIIUDel:α′3 unpaired vs CaMKIIUDel:α3 unpaired	6	6	0.284	40	>0.9999
CaMKIIUDel:α′3 paired vs CaMKIIUDel:α3 paired	6	6	3.488	40	0.0143

^a^Statistical data for each comparison in Figure 5*F*. N1, Sample size of first group; N2, sample size of second group; *t*, value of the *t* distribution.

## Results

### Artificial aversive training induces a potentiation PDP in KCs

**Figure 1.**
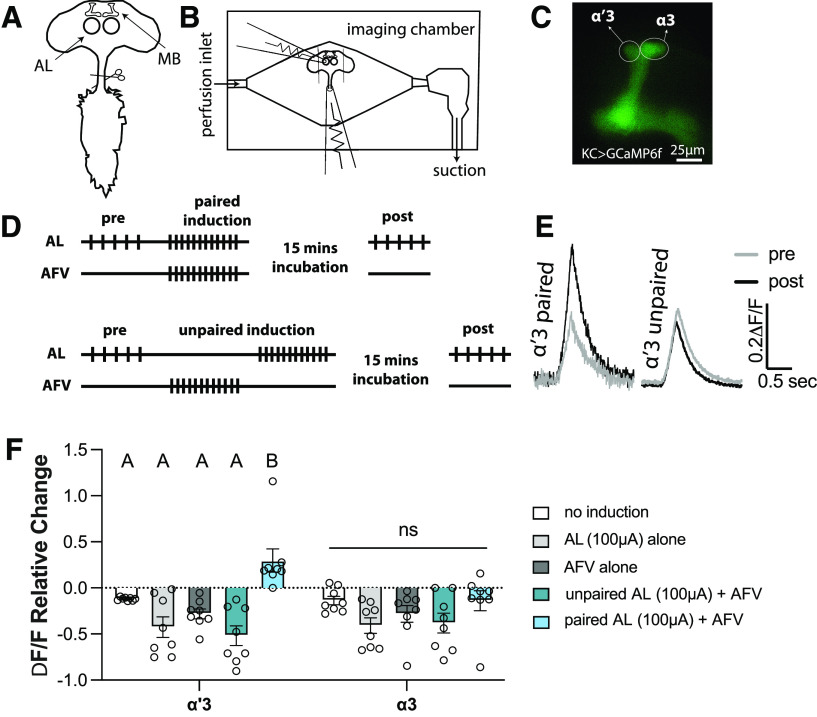
Simultaneous activation of the odor and the electric shock pathways induces an enhancement PDP in the KCs in the MB α′3 compartment. ***A***, Schematic of the dissected adult fly's CNS showing the MBs and the ALs in the central brain and the ventral nerve cord. The ventral nerve cord is cut at the base of the cervical connective to free the AFV. ***B***, Schematic of the imaging chamber showing the placement of the first electrode on the AL, and the second electrode on the free end of the AFV. ***C***, Representative image of the MB ipsilateral to the stimulated AL. The calcium indicator GCaMP6f is expressed in the KCs (driven by *VT030559-GAL4*). Circles represent the analyzed ROIs surrounding the α′3 and the α3 compartments. Scale bar, 25 µm. ***D***, Paired and unpaired induction protocols. Top, In the paired induction, the ipsilateral AL is activated by 5 stimulation trains with 15 s intertrain interval followed by 1 min rest (pre-induction). Twelve trains of stimulations are then delivered to both the AL and the AFV simultaneously (induction). The brain is then rested for 15 min before being tested by 5 trains of AL stimulations like those applied during the pre-induction (post-induction). Bottom, The unpaired induction: same as paired induction, except that the AL and the AFV stimulations are separated by 30 s during the induction stage. The stimulation train is 20 pulses at 100 Hz; each pulse is 1 ms with 9 ms interpulse interval. AL stimulation strength: 100 µAmps. AFV stimulation strength: 0.5-1 mAmps. ***E***, Example of the KC calcium response in the α′3 compartment pre (gray) and post (black) paired and unpaired inductions. ***F***, Mean relative change of the calcium responses calculated as (mean post-responses – mean pre-responses)/(mean pre-responses) in the α′3 (left) and the α3 (right) compartments after no induction (white), AL activation alone (light gray), AFV activation alone (dark gray), unpaired AL+AFV induction (green), and paired AL+AFV induction (blue). Data are mean ± SEM. Two-way ANOVA (α = 0.05; *n* = 8 in each condition): lobe effects, *F*_(1,70)_ = 1.096, *p* = 0.2987; induction effects, *F*_(4,70)_ = 11.51, *p* < 0.0001. Sidak *post hoc* tests: in the α′3 compartment: A versus A, *p* > 0.05; B versus A, *p* ≤ 0.05; *t*_(70)_ {3.23, *p* = 0.015; 5.6, *p* < 0.0001; 4.48, *p* = 0.0002; 6.319, *p* < 0.0001} for {paired vs no induction; paired vs AL alone; paired vs AFV alone; paired vs unpaired}, respectively. No statistical significance across conditions in the α3 compartment, *p* > 0.05; *t*_(70)_ = {0.0237, *p* > 0.9999; 2.093, *p* = 0.2785; 1.105, *p* = 0.9218; 1.884, *p* = 0.4094} for {paired vs no induction; paired vs AL alone; paired vs AFV alone; paired vs unpaired}, respectively.

Previous studies had demonstrated that enhanced calcium responses similar to those reported in the MB *in vivo* after aversive training can be achieved in dissected brains (Wang et al., 2008; Tomchik and Davis, 2009; Ueno et al., 2013). To investigate whether this plasticity recapitulates the formation of aversive memory, and to optimize our protocol, we dissected the CNS (brain with attached ventral nerve cord) of 4- to 8-d-old mated female flies expressing the Ca^2+^ indicator GCaMP6f in the MBs using the KC driver (*VT030559*-*GAL4*). Dissected brains were pinned in an imaging bath chamber, and the cervical connective toward the base of the ventral nerve cord was cut to allow electrical stimulation of the AFV connecting the ventral nerve cord to the brain (Fig. 1*A*). We used glass suction electrodes to stimulate the AL projection neurons and the AFV to activate the odor pathway and the electric shock pathway, respectively (Fig. 1*B*). Given that *in vivo* aversive STM formation is correlated with enhanced calcium responses in the α′3 MB compartment but not in the α3 compartment (Krashes et al., 2007; Wang et al., 2008; Cervantes-Sandoval et al., 2013; Zhang et al., 2019), we decided to focus our calcium imaging on these two regions (Fig. 1*C*). We found that both α′3 and α3 compartments respond only to ipsilateral AL stimulation, but AFV stimulation could generate a calcium response in both compartments of both MBs. Therefore, we imaged the MB ipsilateral to the stimulated AL.

To induce the formation of an aversive STM trace, we paired 12 trains of stimulation of both the AL electrode and the AFV electrode, thus activating the CS and the US pathways at the same time (paired induction). As controls, we repeated the same induction paradigm but separated the AL stimulation from the AFV stimulation by 30 s (unpaired induction) or omitted the AFV stimulation (AL alone induction) or omitted the AL stimulation (AFV alone induction) or simply allowed the brain to rest for 15 min (no induction). We compared the change in calcium response to AL stimulation before induction and 15 min after induction (Fig. 1*D*). We predicted that, if this *ex vivo* model truly recapitulated short-term aversive training, only the paired induction should produce an enhancement of the calcium responses in the α′3 and none of the stimuli should potentiate α3 (Krashes et al., 2007; Wang et al., 2008; Davis, 2011; Cervantes-Sandoval et al., 2013). Indeed, this was the result we obtained (Fig. 1*E*,*F*). Interestingly, we noticed that the repetitive unpaired activation of KCs results in suppression of their calcium responses (Fig. 1*F*). This is reminiscent of desensitization or habituation; a nonassociative plasticity/memory described as a decrement in neural responses to uninteresting, frequently encountered stimuli whose predictive value is negligible (Wilson and Linster, 2008; Wilson, 2009). These nonassociative processes have been documented in insects (Cho et al., 2004; Das et al., 2011; Semelidou et al., 2018), rodents (Wilson, 1998; Best and Wilson, 2004), and humans (Ferdenzi et al., 2014; Pellegrino et al., 2017).

### Dopamine replaces the artificial US stimulus but does not replace the CS

**Figure 2.**
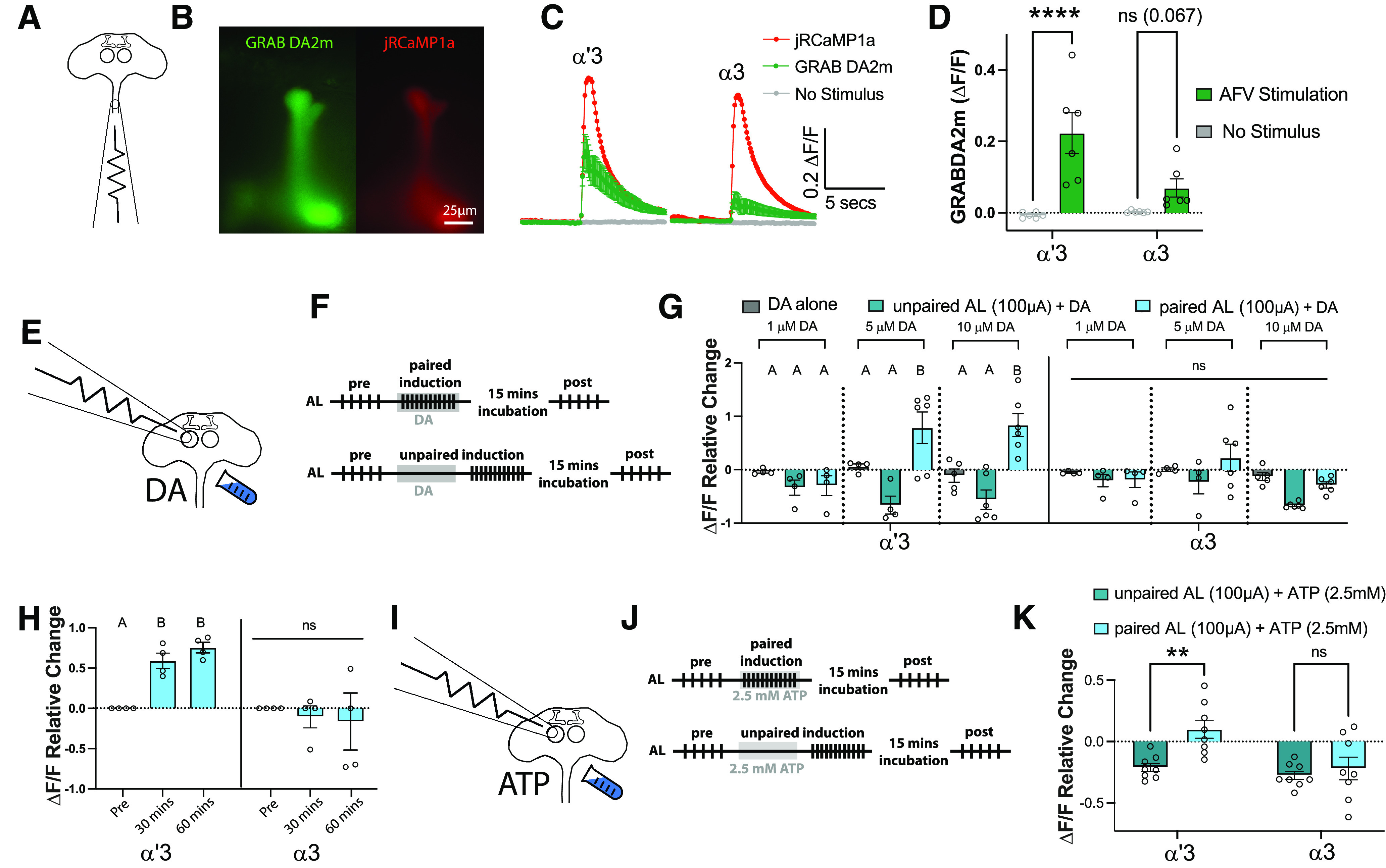
Electric shock pathway activation can be replaced by either dopamine perfusion or activation of dopaminergic neurons. ***A***, Schematic of the dissected adult CNS showing the placement of one electrode to activate the AFV. ***B***, Representative images showing the simultaneous expression of *UAS-GRAB DA2m* (left; green) and *UAS-jRCaMP1a* (right; red) in the same neurons in the MB (driven by *VT030559-GAL4*). Scale bar, 25 µm. ***C***, Time course of GRAB DA2m responses in the α′3 and α3 compartments on AFV stimulation (green) or no stimulus (gray). Traces represent mean ΔF/F ± SEM across the 6 flies tested. In each compartment, a representative jRCaMP1a response is shown (red) as a reference for neural activity on AFV stimulation. ***D***, Quantification of the GRAB DA2m responses in ***C***. Two-way ANOVA (α = 0.05; *n* = 6 in each condition): lobe effects, *F*_(1,20)_ = 3.759, *p* = 0.0668; stimulation effects, *F*_(1,20)_ = 29.52, *p* < 0.0001. Sidak *post hoc* tests: in the α′3 compartment, ****t_(20)_ = 5.411, *p* < 0.0001; in the α3 compartment, ^ns^*t*_(20)_ = 2.273, *p* = 0.0673. ***E***, Schematic of the AL+DA induction protocol, showing electrode placement above the AL, and perfusion of dopamine. ***F***, Paired and unpaired induction protocols. Top, In the paired induction, the ipsilateral AL is activated by 5 stimulation trains with 15 s intertrain interval followed by resting for 1 min (pre-induction). Twelve trains of stimulations are then applied via the AL electrode, coincident with 60 s of dopamine perfusion. The brain is then rested for 15 min before being tested by 5 trains of AL stimulations like those applied during the pre-induction (post-induction). Bottom, The unpaired induction: same as paired induction, except that the AL stimulation and dopamine perfusion are separated by 30 s during the induction stage. The stimulation train is 20 pulses at 100 Hz; each pulse is 1 ms with 9 ms interpulse interval. AL stimulation strength: 100 µAmps. ***G***, Mean relative change of the calcium responses in the α′3 (left) and the α3 (right) compartments after DA perfusion alone (gray), unpaired AL+DA induction (green), and paired AL+DA induction (blue). Three different DA concentrations were used: 1 μm (left), 5 μm (middle), and 10 μm (right). Data are mean ± SEM. Two-way ANOVA: lobe effects, *F*_(1,68)_ = 2.724, *p* = 0.1034; induction effects, *F*_(8,68)_ = 9.584, *p* < 0.0001. Tukey's *post hoc* results are listed in Table 1. ***H***, Mean relative change of the calcium responses in the α′3 (left) and the α3 (right) compartments before induction and 30 or 60 min after paired AL+ 10 μm DA induction (blue). Data are mean ± SEM. Two-way ANOVA with repeated measures (α = 0.05; *n* = 4 in each condition): lobe effects, *F*_(1,6)_ = 12.67, *p* = 0.0119; time effects, *F*_(1.341,8.045)_ = 2.249, *p* = 0.1719; lobe × time interaction effects, *F*_(2,12)_ = 5.206, *p* = 0.0236. Sidak *post hoc* tests: in the α′3 compartment: A versus A, *p* > 0.05; B versus A, *p* ≤ 0.05; *t*_(3)_ = {6.216, *p* = 0.025; 11.64, *p* = 0.0041; 1.03, *p* = 0.7604} for {pre vs 30 min; pre vs 60 min; 30 min vs 60 min}, respectively. No statistical significance across conditions in the α3 compartment, *p* > 0.05; *t*_(3)_ = {0.7799, *p* = 0.8692; 0.4625, *p* = 0.9657; 0.1993, *p* = 0.9969} for {pre vs 30 min; pre vs 60 min; 30 min vs 60 min}, respectively. ***I***, Schematic of the AL+PPL1 induction protocol, showing electrode placement above the AL, and perfusion of 2.5 mm ATP to activate the P2X_2_ channels expressed in the PPL1 dopaminergic neurons (driven by *TH-LexA*). ***J***, Paired and unpaired induction protocols. Top, In the paired induction, the ipsilateral AL is activated by 5 stimulation trains with 15 s intertrain interval followed by resting for 1 min (pre-induction). We then apply 12 trains of stimulations to the AL electrode, coincident with 60 s of 2.5 mm ATP perfusion. The brain is then rested for 15 min before being tested by 5 trains of AL stimulations like those applied during the pre-induction (post-induction). Bottom, The unpaired induction: same as paired induction, except that the AL stimulation and ATP perfusion are separated by 30 s during the induction stage. The stimulation train is 20 pulses at 100 Hz; each pulse is 1 ms with 9 ms interpulse interval. AL stimulation strength: 100 µAmps. ***K***, Mean relative change of the calcium responses in the α′3 (left) and the α3 (right) compartments after unpaired AL+ATP induction (green), and paired AL+ATP induction (blue). Data are mean ± SEM. Two-way ANOVA (α = 0.05; *n* = 8 in each condition): lobe effects, *F*_(1,28)_ = 9.053, *p* = 0.0055; induction effects, *F*_(1,28)_ = 8.284, *p* = 0.0076. Sidak *post hoc* tests: in the α′3 compartment, **t_(28)_ = 3.446, *p* < 0.0036; In the α3 compartment, ^ns^*t*_(28)_ = 0.624, *p* = 0.7862.

Multiple behavioral and *in vivo* imaging studies have shown that dopaminergic neurons encode the US valence information in the MB, with PPL1s providing aversive reinforcement and PAMs providing appetitive reinforcement (Schwaerzel et al., 2003; Riemensperger et al., 2005; Schroll et al., 2006; Kim et al., 2007; Claridge-Chang et al., 2009; Mao and Davis, 2009; Aso et al., 2010, 2012, 2014b; Pech et al., 2013; Yamagata et al., 2016; Handler et al., 2019). These studies suggest that the US alone is sufficient to evoke dopamine release. However, mechanistic studies using the LTE paradigm reported no dopamine release after US pathway stimulation alone. Strong dopamine release was only seen after coincident activation of both the CS and the US pathways, and it was concluded that dopamine release is downstream of the CS+US coincidence and does not encode the primary US information (Ueno et al., 2017, 2020). To directly address this discrepancy, we expressed a G-protein-coupled receptor activation-based dopamine sensor (*GRAB_DA_*) (Sun et al., 2020) in the KCs. We then dissected the fly's CNS and activated the US pathway by stimulating the AFV. We also expressed the calcium indicator jRCaMP1a in the KCs to use as a reference for the strength of activation (Fig. 2*A*,*B*). We observed a robust dopamine release in the α′3 and a much weaker release onto the α3 compartment in response to the same AFV stimulation used in our induction experiments (Fig. 2*C*,*D*). This result shows that dopamine release occurs in response to the US stimulus alone and does not require CS+US coincidence.

We next asked whether dopamine perfusion can replace electrical stimulation of the AFV. We paired electrical stimulation of the AL with perfusion of different concentrations (1, 5, and 10 μm) of dopamine and analyzed the KC response to AL stimulation before and after the pairing (Fig. 2*E*,*F*). Similar to the plasticity seen after AL+AFV induction, we found that only the paired AL+ DA (5 or 10 μm) induction induced an enhancement PDP in the α′3; pairing with 1 μm DA was not sufficient to induce PDP (Fig. 2*G*; for detailed statistical data for the *post hoc* analysis, see Table 1). Also, in agreement with our observation that dopamine release in α3 was very weak compared with the α′3 compartment on AFV stimulation, our protocol did not induce *ex vivo* PDP in the α3 compartment (Fig. 2*G*). We also found that PDP (induced by AL + 10 μm DA) lasts for at least 1 h after induction (Fig. 2*H*).

Although these results supported a PDP model with direct dopamine modulation of KCs, we were concerned with the lack of synaptic specificity in the AL+DA induction, as dopamine was perfused onto the whole preparation. To address this concern, we repeated the experiment but replaced dopamine perfusion with activation of the dopaminergic neurons in the PPL1 cluster, which carry the negative valence US information to the MB (Schroll et al., 2006; Claridge-Chang et al., 2009; Mao and Davis, 2009; Aso et al., 2010, 2012; Burke et al., 2012; Aso and Rubin, 2016). We expressed the ATP-gated channel *P2X_2_* (Yao et al., 2012) in the PPL1 dopaminergic cluster using the *TH-LexA* driver. We paired AL stimulation with application of 2.5 mm ATP to activate PPL1s (Fig. 2*I*,*J*). Again, we observed an enhancement PDP in the α′3 but not in the α3 compartment (Fig. 2*K*).

Together, our findings agree with previous behavioral studies and *in vivo* imaging studies and show that dopamine is released in response to the US alone and carries US valence information to the MB to allow associative learning. Potentiation PDP induced in our *ex vivo* paradigm also localizes to α′ cells only, congruent with the memory traces reported *in vivo* with aversive STM.

### PDP *ex vivo* plasticity is input-specific

**Figure 3.**
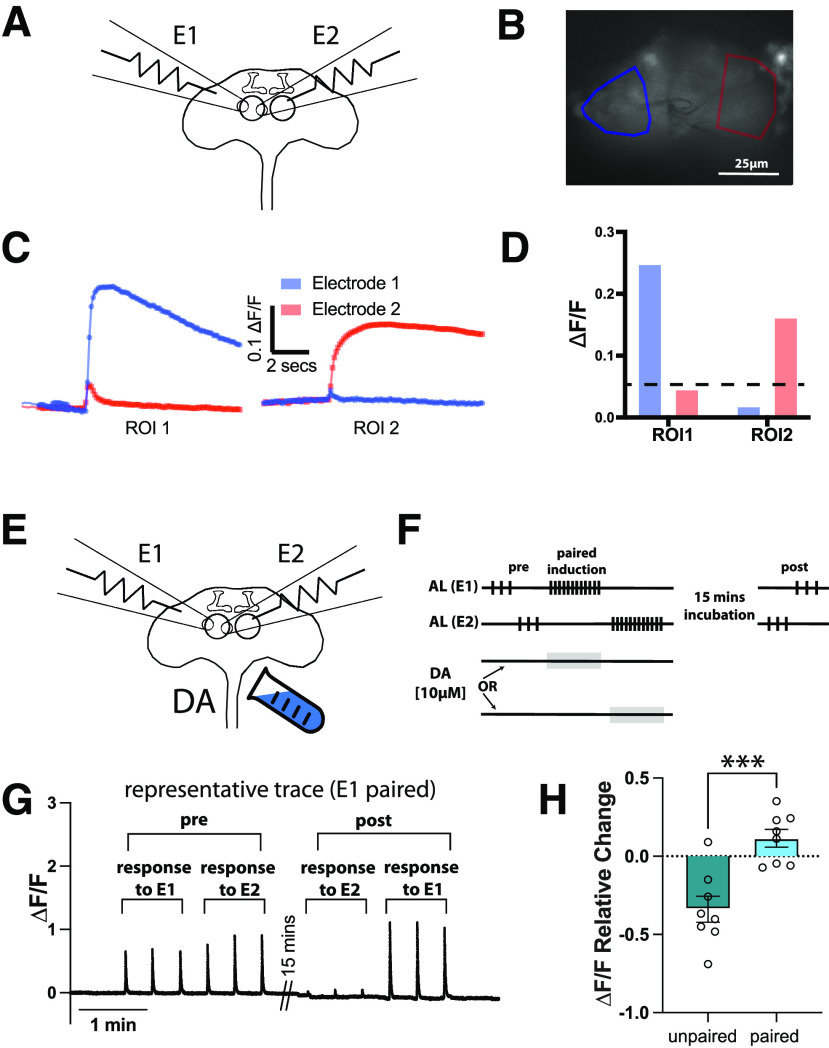
PDP is CS-pathway input-specific. ***A***, Schematic of the dissected adult brain showing the placement of two electrodes (E1 and E2) onto the surface of the same AL. ***B***, Basal calcium signals in the AL projection neurons (genotype: *w-;GH146-GAL4/*+*;UAS-GCaMP6f/*+) showing two ROIs highlighted in blue (ROI near E1) and red (ROI near E2). ***C***, Calcium responses in each ROI to stimulation from E1 (blue trace) or E2 (red trace). ***D***, Quantification of ***C***. Dashed line is plotted at ΔF/F = 0.05. ***E***, Same schematic as in ***A*** but with dopamine perfusion. ***F***, Induction protocol. The ipsilateral AL is activated by 3 stimulation trains with 15 s intertrain interval followed by resting for 1 min (pre-induction). Twelve trains of stimulations to electrode 1 (E1) are then delivered, followed by a 30 s rest, then 12 trains of stimulations to electrode 2 (E2). Dopamine [10 μm] is perfused in the chamber for 60 s paired with either E1 or E2 stimulation. The brain is then rested for 15 min before being tested by 3 trains of E2 stimulation followed by 3 trains of E1 stimulation (post-induction). ***G***, Representative trace of a prep in which dopamine perfusion is paired with E1 stimulations. ***H***, Mean relative change of the calcium responses in the α′3 compartment. Data are mean ± SEM. Unpaired *t* test, two-tailed; *n* = 8: ***t_(14)_ = 4.505, *p* = 0.0005.

Associative memory is CS-specific. A fly that is aversively trained against an odor shows aversive behavior to that odor and closely similar odors only but does not generalize the aversion to all odors (Barth et al., 2014). Olfactory information is sparsely transmitted to KC dendrites, with the identity of the odor being determined by population coding (Marin et al., 2002; Perez-Orive et al., 2002; Wong et al., 2002; Murthy et al., 2008; Turner et al., 2008; Honegger et al., 2011). To mimic the application of two different odors to the fly brain, we placed two suction glass electrodes onto the same AL and spaced them as far as possible from each other to maximize the probability that each electrode stimulates a distinct subset of projection neurons. Theoretically, a certain degree of overlap between the two subsets could be allowed as long as the two activated populations are sufficiently distinct. To test whether the two electrodes activated distinct populations of projection neurons, we expressed the calcium indicator (GCaMP6f) in the projection neurons and divided the AL into multiple ROIs, asking whether the pattern of responses was equivalent for the two electrodes (Fig. 3*A*,*B*). We found that spacing resulted in activation of distinct populations of projection neurons, allowing us to provide two hypothetical odors to the MB (Fig. 3*C*,*D*).

We then asked whether the observed *ex vivo* PDP is specific to the olfactory input activated during induction. To challenge the olfactory-input specificity of our preparation even more, we used the less specific induction method, AL+DA, in which dopamine is impartially perfused to the whole brain to eliminate any specificity on the US pathway side (Fig. 3*E*). We recorded KC calcium responses to both electrode stimulations before induction (pre). Then, we delivered a train of stimulations through electrode 1, followed by a train of stimulations through electrode 2. Dopamine perfusion started 5 s before the stimulation train for only one of the two electrodes (paired) and stopped at the last pulse of the train, while normal HL3 saline was perfused during the activation of the other electrode (unpaired). We then allowed the brain to rest for 15 min and tested the KC response to electrode 2 first, then to electrode 1 to eliminate any bias because of the order of stimulation (Fig. 3*F*). An increased calcium response was observed in α′3 after activation of the paired subset compared with the unpaired one (Fig. 3*G*,*H*). This result demonstrates that even with the broader bath-dopamine induction paradigm, the plasticity achieved in our paradigm is specific to the CS input activated during *ex vivo* training.

### Artificial aversive training induces a suppression PDP in α′3 MBONs

**Figure 4.**
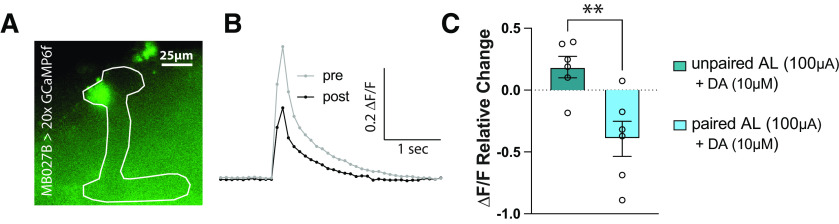
α′3 MBON dendrites show a suppression PDP. ***A***, Representative image of the GCaMP6f signal expressed in the α′3 MBONs using the *MB027B* splitGAL4 line. The MB is outlined in white, showing the localization of the analyzed dendritic signal in the α′3 compartment. Scale bar, 25 µm. ***B***, Representative trace of the α′3 MBON responses before and after the AL+DA induction as described in Figure 2*F*. ***C***, Mean relative change of the calcium responses in the MBONs' dendrites in the α′3 compartment. Data are mean ± SEM. Unpaired *t* test, two-tailed; *n* = 6: **t_(10)_ = 3.495, *p* = 0.0058.

While the α′ branches of the KCs are potentiated in response to an aversively trained odor in intact animals, responses in their postsynaptic MBONs are suppressed (Sejourne et al., 2011; Owald and Waddell, 2015; Owald et al., 2015; Zhang et al., 2019). To eliminate the possibility that our paradigm potentiates memory-relevant KCs via a memory-irrelevant epiphenomenon, we needed to demonstrate that our *ex vivo* training could produce a suppression PDP in MBONs. Therefore, we repeated the same AL+DA induction described previously (Fig. 2*F*), but this time we expressed *20xGCaMP6f* with the *MB027b split-GAL4* driver to examine the responses in the α′3 MBONs (MBON-α′3ap and MBON-α′3m) (Tanaka et al., 2008; Sejourne et al., 2011; Aso et al., 2014a) (Fig. 4*A*). We found that the α′3 MBON response to AL stimulation is suppressed after our artificial learning paradigm (Fig. 4*B*,*C*), agreeing with *in vivo* results. These findings show that both the enhancement and suppression PDPs achieved in our paradigm are learning-specific and are encoded as enhancement in the presynaptic KCs and a suppression in the postsynaptic MBONs.

### The 3′UTR mRNA of *CaMKII* is important for *ex vivo* PDP

We next turned our attention to exploring the utility of this paradigm for understanding memory formation, choosing problems at several different levels of analysis: molecular components of the memory machinery, organizational principles of the circuitry, and the role of brain state in gating plasticity. At the molecular level, previous studies have demonstrated that CaMKII is important for synaptic plasticity and memory formation in many species (Kelleher et al., 2004; Giese and Mizuno, 2013), including *Drosophila melanogaster* (Griffith et al., 1993; Koh et al., 1999; Ashraf et al., 2006; Malik et al., 2013; Mitchell et al., 2021). Recently, we found that the long 3′UTR region of *CaMKII* mRNA is responsible for the activity-dependent synthesis of CaMKII in presynaptic terminals at the larval neuromuscular junction (Kuklin et al., 2017) and for the basal accumulation of axonal CaMKII protein in MB (Chen et al., 2022). We hypothesized that the loss of the 3′UTR would impair associative plasticity by decreasing synaptic CaMKII protein levels and disrupting the signaling machinery that is triggered by the CS+US coincidence. We tested this idea and used our *ex vivo* PDP preparation to gain insight into whether this effect is upstream or downstream of the CS+US coincidence.

**Figure 5.**
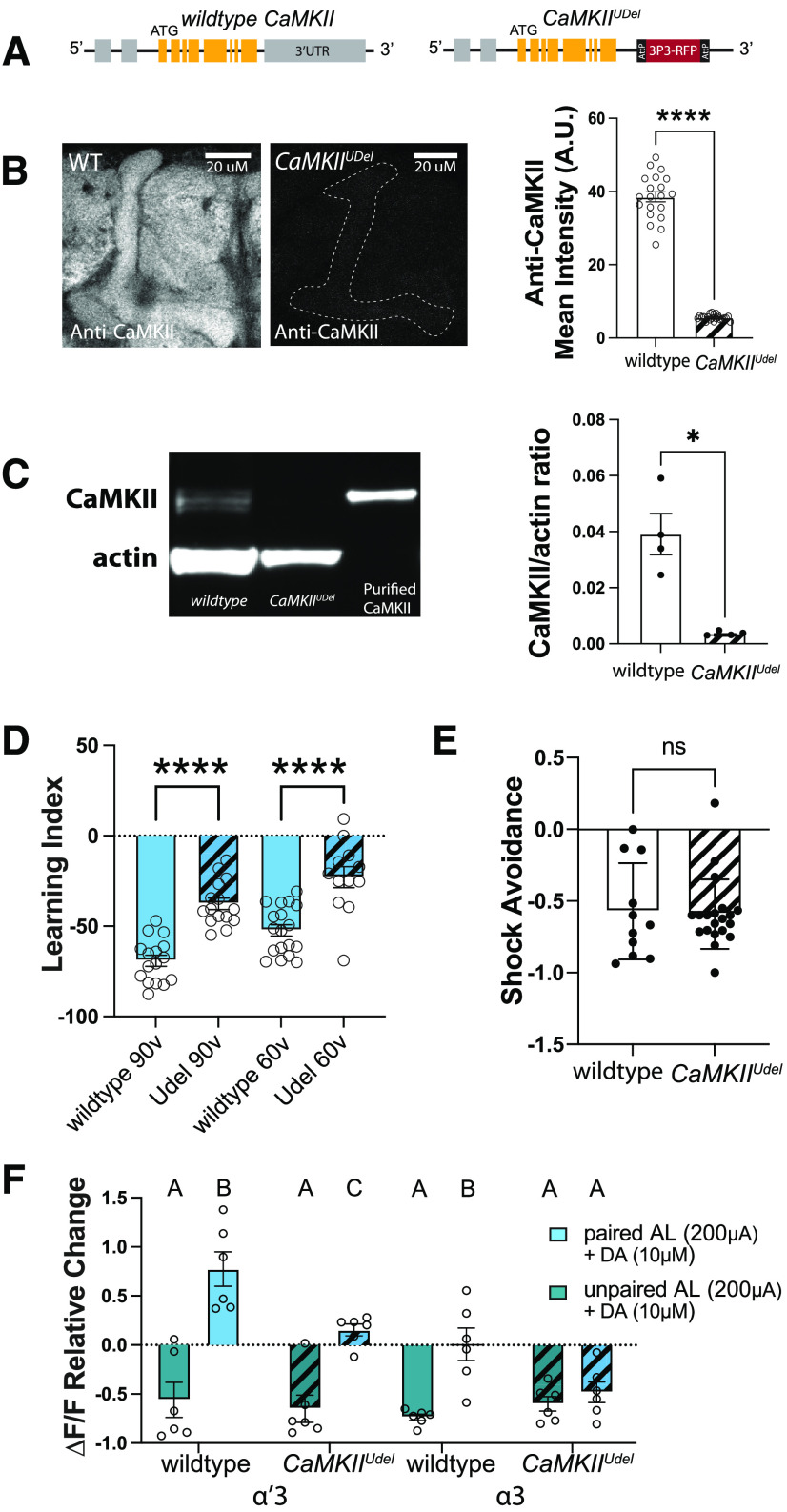
The 3′UTR region of *CaMKII* is important for PDP formation. ***A***, Schematic of WT (left) and *CaMKII^Udel^*, an allele in which the 3′UTR has been replaced with an RFP marker using CRISPR/Cas9 (right). ***B***, Left, Representative immunostaining images showing CaMKII protein levels in the MB in WT *CaMKII* flies and in *CaMKII^Udel^* flies. Dotted line indicates position of the MB. Scale bar, 20 µm. Right, Quantification of CaMKII levels (mean ± SEM). Unpaired *t* test, two-tailed; *n* = 20 WT flies and 22 *CaMKII^Udel^* flies: ****t_(40)_ = 24.81, *p* < 0.0001. ***C***, Left, Western blot showing CaMKII and actin levels in WT or *CaMKII^Udel^* adult female brains and in purified CaMKII samples. Right, Quantification of CaMKII immunoreactivity (mean ± SEM). Mann–Whitney test; *n* = 4 in each condition; sum of ranks in WT group is 26; sum of ranks in *CaMKII^Udel^* group is 10: *p = 0.0286. ***D***, Learning index of WT and *CaMKII^Udel^* flies after training with 90 or 60 V. Data are mean ± SEM. Two-way ANOVA: punishment voltage effects, *F*_(1,57)_ = 18, *p* < 0.0001; 3′UTR effects, *F*_(1,57)_ = 67.57, *p* < 0.0001. Sidak *post hoc* tests: when punishment is 60 V, ****t_(57)_ = 5.516, *p* < 0.0001; when punishment is 90 V, ****t_(57)_ = 6.122, *p* < 0.0001. ***E***, WT and *CaMKII^Udel^* flies can avoid electric shock. Data are mean ± SEM. Unpaired *t* test; data collected across 11 trials of the WT group and 20 trials of the *CaMKII^Udel^* group; number of flies per group ranged between 21 and 47 with an average of 29 flies per group: ^ns^*p* = 0.8518. ***F***, Mean relative change of the calcium responses in the α′3 and the α3 compartments after unpaired AL+DA induction (green) and paired AL+DA induction (blue) in WT flies or *CaMKII^Udel^* flies. Data are mean ± SEM. Three-way ANOVA; *n* = 6 in each condition: lobe effects, *F*_(1,40)_ = 17.52, *p* = 0.0002; induction effects, *F*_(1,40)_ = 68.43, *p* < 0.0001; 3′UTR effects, *F*_(1,40)_ = 8.625, *p* = 0.0055; 3′UTR × lobe interaction effects, *F*_(1,40)_ = 0.9631, *p* = 0.3323; 3′UTR × induction interaction effects, *F*_(1,40)_ = 10.16, *p* = 0.0028; lobe × induction effects, *F*_(1,40)_ = 12.25, *p* = 0.0012; 3′UTR × lobe × induction interaction effects, *F*_(1,40)_ = 0.07388, *p* =0.7872. Sidak *post hoc* results are listed in Table 2. Striped bars represent the *CaMKII^Udel^* genotype.

First, we asked whether loss of the 3′UTR of *CaMKII* mRNA affects CaMKII levels. We used animals in which the *CaMKII* gene was engineered using CRISPR/Cas9 to lack the 3′UTR (*CaMKII^Udel^*, Fig. 5*A*) (Chen et al., 2022). Immunostaining to quantify CaMKII protein levels in the MB neuropil in both WT and *CaMKII^UDel^* showed that CaMKII levels are specifically decreased in synaptic regions (Fig. 5*B*). We also used Western blotting to quantify total CaMKII levels and found a substantial decrease, normalized to actin, in *CaMKII^UDel^* flies compared with WT (Fig. 5*C*). This is in agreement with a previous study which found that mice expressing a mutant form of CaMKII lacking the 3′UTR show decreased levels of CaMKII in the dendritic, but not the somatic, region of hippocampal neurons (Miller et al., 2002).

We then tested the impact of the 3′UTR deletion on aversive STM. We found that *CaMKII^UDel^* flies showed a significant impairment in immediate memory performance compared with WT flies (Fig. 5*D*). This impairment was not because of sensorimotor dysfunctionalities as both WT and *CaMKII^UDel^* flies respond to electric shock (Fig. 5*E*). We then tested the effect of the *CaMKII* mRNA 3′UTR deletion on potentiation PDP in KCs. We used the previously described AL+DA induction protocol and examined KC responses to AL stimulation both before and 15 min after either paired or unpaired induction. In the α′3 compartment, although both WT and *CaMKII^UDel^* genotypes showed statistically significant PDP relative to the unpaired induction, the PDP in the WT flies was >5 times stronger than in the *CaMKII^UDel^* flies (Fig. 5*F*; for detailed statistical data for the *post hoc* analysis, see Table 2). We also noticed that the change in signal in the α3 compartment in WT flies after paired induction was statistically different from the unpaired induction. However, this does not translate into formation of PDP in that compartment as this change was not different from zero (one-sample *t* test, *p* = 0.947). This is likely because of a technical difference as the AL input stimulation in this experiment was stronger (200 µAmps vs 100 µAmp in previous experiments), which led to a stronger suppression after the unpaired AL stimulation in the unpaired induction (see also below). These results show that the 3′UTR mRNA of *CaMKII* is important for memory and its loss impairs learning-induced plasticity. It also indicates that the effect on the learning circuit is downstream of the CS+US coincidence and does not block the CS+US coincidence detection machinery itself.

### Stronger inputs to the MB can recruit the α3 compartment into the STM circuit

**Figure 6.**
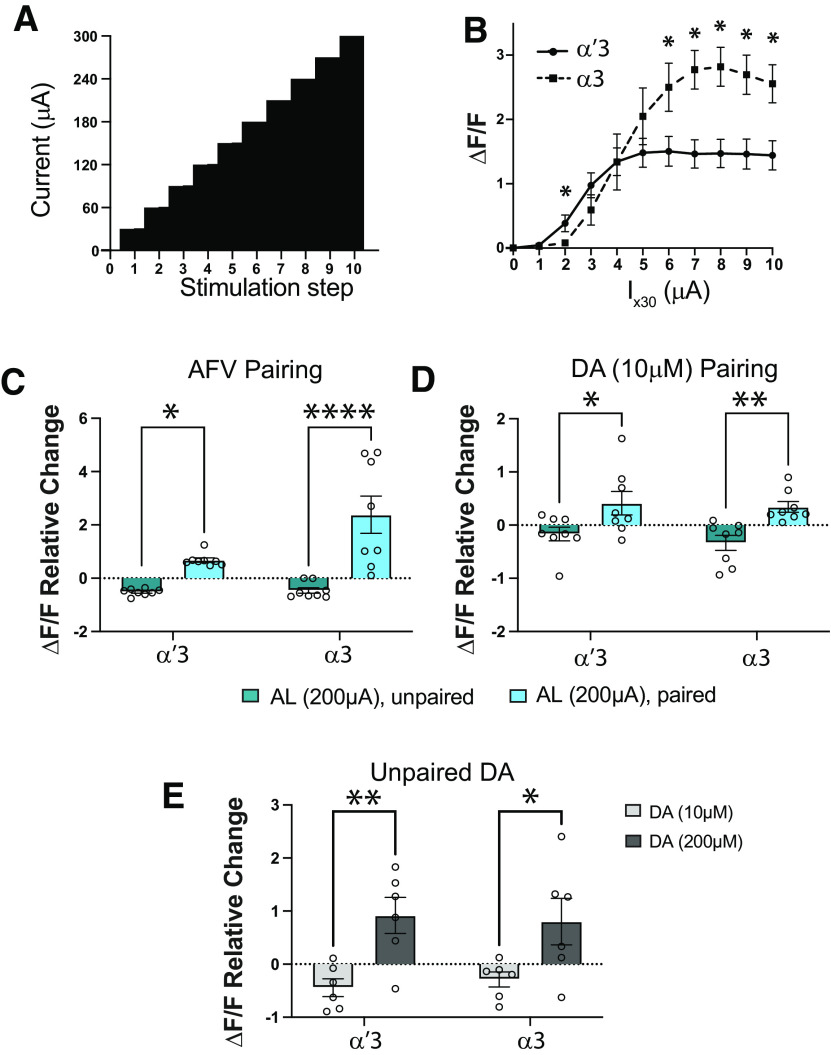
Increasing CS or US pathway activation recruits the α3 compartment to the PDP circuit. ***A***, Design of the AL ramp. AL input stimulation is increased by 30 µAmps every 30 s. ***B***, Unpaired calcium responses to AL stimulations in the α′3 and α3 compartments showing mean ± SEM. Unpaired *t* test; *n* = 8, *p < 0.05: *t*_(14)_ = {1.873, *p* = 0.082; 2.304, *p* = 0.0371; 1.254, *p* = 0.23; 0.00567, *p* = 0.99555; 1.143, *p* = 0.2722; 2.267, *p* = 0.0397; 3.517, *p* = 0.00342; 3.6, *p* = 0.002898; 3.218, *p* = 0.006193; 2.979, *p* = 0.009959} for comparisons between α′3 and α3 responses to {30; 60; 90; 120; 150; 180; 210; 240; 270; 300} µAmps AL stimulation, respectively. ***C***, ***D***, Mean relative change of the calcium responses in the α′3 and the α3 compartments after the unpaired (green) or paired (blue) AL+AFV induction in ***C*** and AL+DA induction in ***D*** when the AL input stimulation is increased to 200 µAmps. Data are mean ± SEM. ***C***, Two-way ANOVA (α = 0.05; *n* = 8 in each condition): lobe effects, *F*_(1,28)_ = 6.078, *p* = 0.0201; induction effects, *F*_(1,28)_ = 32.08, *p* < 0.0001; lobe × induction interaction effects, *F*_(1,28)_ = 5.338, *p* = 0.0285. Sidak *post hoc* tests: in the α′3 compartment, *t_(28)_ = 2.372, *p* = 0.049; in the α3 compartment, ****t_(28)_ = 5.639, *p* < 0.0001. ***D***, Two-way ANOVA (α = 0.05; *n* = 8 in each condition): lobe effects, *F*_(1,28)_ = 0.5793, *p* = 0.4530; induction effects, *F*_(1,28)_ = 16.36, *p* = 0.003; lobe × induction interaction effects, *F*_(1,28)_ = 0.1038, *p* = 0.74985. Sidak *post hoc* tests: in the α′3 compartment, *t_(28)_ = 2.656, *p* = 0.0256; in the α3 compartment, **t_(28)_ = 3.112, *p* = 0.0085. ***E***, Mean relative change of the calcium responses in the α′3 and the α3 compartments after unpaired 10 or 200 μm DA perfusion alone, stimulation at 100 µAmps. Two-way ANOVA (α = 0.05; *n* = 6 in each condition): lobe effects, *F*_(1,20)_ = 0.005471, *p* = 0.9418; concentration effects, *F*_(1,20)_ = 16.88, *p* = 0.0005; lobe × concentration interaction effects, *F*_(1,20)_ = 0.2026, *p* = 0.6574. Sidak *post hoc* tests: in the α′3 compartment, **t_(20)_ = 3.223, *p* = 0.0085; in the α3 compartment, *t_(20)_ = 2.586, *p* = 0.035.

At the circuit level, it has been hypothesized that memory in flies, like humans (McClelland et al., 1995; Dudai, 2012), undergoes systems consolidation: initial potentiation of the α′β′ lobes with a time-dependent transfer of potentiation to αβ. The MB α′3 and α3 compartments are adjacent to each other. Both respond to odor (Turner et al., 2008) and AL stimulation (Fig. 6*B*), and both use the same coincidence detector, *rutabaga* (Livingstone et al., 1984; Levin et al., 1992; Mao et al., 2004; Gervasi et al., 2010). This raises the question of how the two compartments are able to play different roles in the memory circuit and why only the α′β′ cells show immediate PDP both *ex vivo* and *in vivo*. To determine whether this might be because of differences in the intrinsic properties of the two classes of cells, we examined the basal responses of both compartments to a ramp of AL stimulations without any pairings (Fig. 6*A*). We found that α′3 axons are recruited first, with very weak AL stimulation, while α3 only starts responding at higher stimulation strengths (Fig. 6*B*). There was also a distinct difference in the maximum response levels: α′3 compartment responses plateau at a much lower level than α3 (Fig. 6*B*), likely explained by the fact that the α3 compartment receives more axons than α′3 (F. Li et al., 2020). Previous studies reported that odors activate a small percentage of KCs (∼5%-12%), and that the odor responses in α′β′ cells are stronger than those of αβ cells (Turner et al., 2008; Inada et al., 2017). This suggests that actual odor encoding is similar to the weak AL stimulation used in our experiments (100 µAmp), recruiting only α′3 but insufficient to recruit α3. We hypothesized that an increase in the AL stimulation intensity during *ex vivo* induction might be sufficient to recruit α3 and allow PDP in that compartment as well. We repeated both AL+AFV and AL+DA inductions, but this time with a 200 µAmp AL stimulation intensity. Indeed, under these conditions, we found that PDP occurred in both the α′3 and α3 compartments (Fig. 6*C*,*D*).

This result agrees with predictions from our previously published theoretical model of the associative learning circuit in which the CS+US coincidence triggers a recurrent loop between KCs and dopaminergic neurons, which increases dopamine release only onto cells receiving both inputs together (Adel and Griffith, 2021). The weak response of the α3 compartment to weak AL stimulation is subthreshold for the MB coincidence detector, and insufficient for forming PDP in α3. Stronger AL stimulation puts α3 over threshold and triggers the coincidence detector in α axons, thus increasing dopamine gain and forming PDP/memory.

Our model further predicts that there should be a certain dopamine concentration above which dopamine alone is sufficient to potentiate both α′3 and α3 compartments without any need for pairing, as this hypothetical high dopamine concentration bypasses the need for CS+US coincidence detector activation of the gain control machinery that increases local dopamine release. To test this prediction, we applied either 10 μm dopamine (used in our previous experiments) or a high dopamine concentration (200 μm) to the dissected brains without pairing with AL stimulation. Indeed, 200 μm dopamine alone was sufficient to potentiate both α′3 and α3 responses to AL stimulation (100 µAmps) 15 min after dopamine application (Fig. 6*E*). A similar effect of high dopamine was reported by Ueno et al. (2013) but interpreted differently. We show that pairing-independent plasticity resulting from very high dopamine, while possibly implemented by the same machinery responsible for associative learning, does not truly represent a memory trace as it lacks specificity to CS-activated synapses, likely representing a generalized potentiated state of the MB. In contrast, the PDP seen with lower dopamine concentrations relies on an interplay between the CS and US signals to gate dopamine gain only at the synapses activated by the CS during training.

### PDP is blocked by sleep deprivation and rescued by rebound sleep

**Figure 7.**
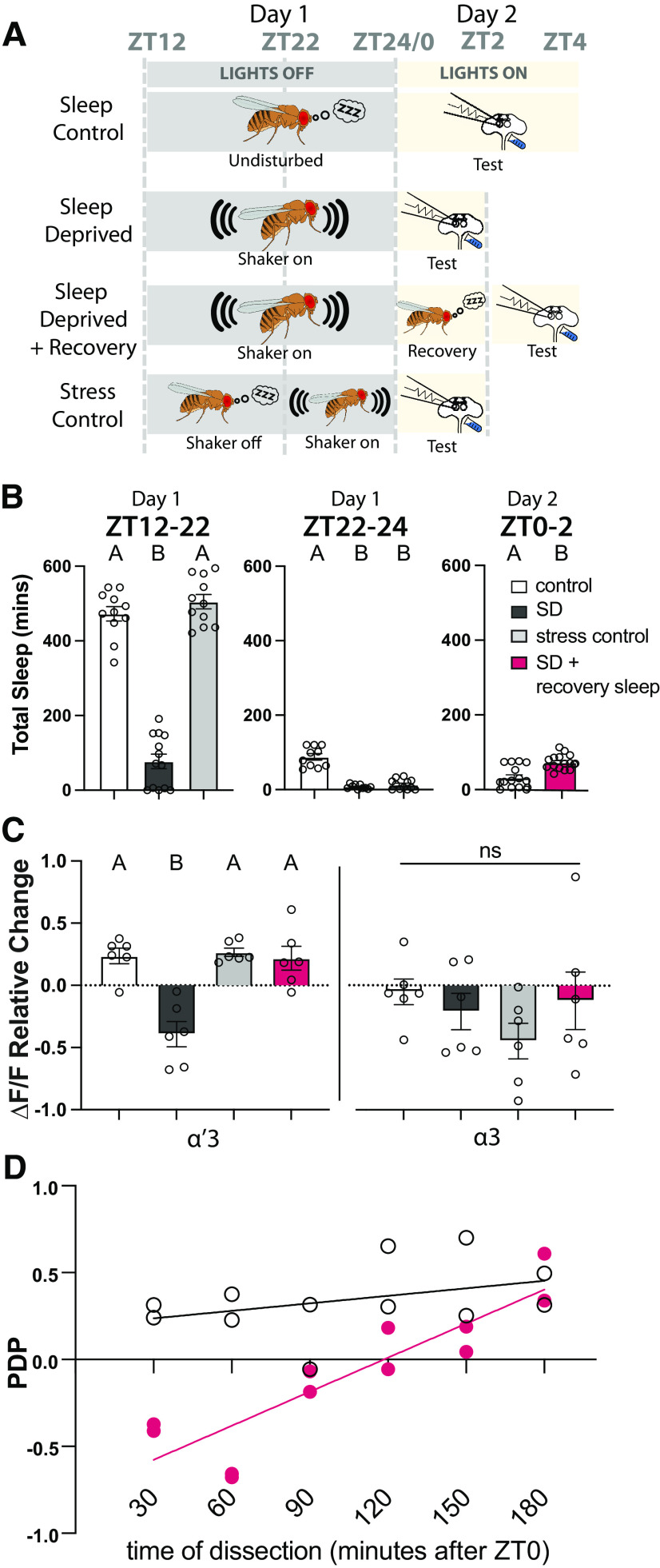
Sleep deprivation blocks PDP and rebound sleep recovers it. ***A***, Schematic of the different sleep groups: Sleep Control, flies are undisturbed during night and tested between ZT0 and ZT4 the next day; Sleep-deprived, flies are kept on the shaker during between ZT12 and ZT24 and then tested between ZT0 and ZT2 the next day; Sleep-deprived + Recovery, same as Sleep-deprived, but flies were allowed to recover sleep between ZT0 and ZT2 and then tested between ZT2 and ZT4; Stress Control, flies are undisturbed during the night between ZT12 and ZT22 and then the shaker was turned on between ZT22 and ZT24 before testing between ZT0 and ZT2 the next day. Testing was done by the AL+DA induction described in Figure 2*F*. ***B***, Total sleep of the different groups in the different ZT windows. Data are mean ± SEM. One-way ANOVA: in the ZT12:ZT22 comparison, *F*_(2,32)_ = 159, *p* < 0.0001; Tukey's *post hoc* {control vs sleep-deprived: *q*_(32)_ = 20.65, *p* < 0.0001; control vs stress control: *q*_(32)_ = 1.625, *p* = 0.4916; sleep-deprived vs stress control: *q*_(32)_ = 22.34, *p* < 0.0001). In the ZT22:ZT24 comparison, *F*_(2,29)_ = 66.64, *p* < 0.0001; Tukey's *post hoc* {control vs sleep-deprived: *q*_(29)_ = 14, *p* < 0.0001; control vs stress control: *q*_(29)_ = 14.47, *p* < 0.0001; sleep-deprived vs stress control: *q*_(29)_ = 1.114, *p* = 0.7136). In the ZT0:ZT2 comparison, *t*_(29)_ = 4.534, *p* < 0.0001. ***C***, Mean relative change of the calcium responses in the α′3 (left) and the α3 (right) compartments after paired AL+DA induction. Data are mean ± SEM. Two-way ANOVA (α = 0.05; *n* = 6 in each condition): lobe effects, *F*_(1,40)_ = 10.10, *p* = 0.0029; sleep effects, *F*_(3,40)_ = 7.019, *p* = 0.0007; lobe × sleep interaction effects, *F*_(3,40)_ = 0.9308, *p* = 0.4348. Sidak *post hoc* tests: in the α′3 compartment: A versus A, *p* > 0.05; B versus A, *p* ≤ 0.05; *t*_(40)_ = {0.1592, *p* > 0.9999; 3.475, *p* = 0.0074; 0.1038, *p* > 0.9999; 3.634, *p* = 0.0047; 0.2631, *p* > 0.9999; 3.371, *p* = 0.01} for {sleep control vs stress control; sleep control vs sleep-deprived; sleep control vs sleep rebound; stress control vs sleep-deprived; stress control vs sleep rebound; sleep-deprived vs sleep rebound}, respectively. No statistical significance across conditions in the α3 compartment, *p* > 0.05; *t*_(40)_ = {0.8750, *p* = 0.9468; 2.196, *p* = 0.1871; 0.4001, *p* = 0.9991; 1.321, *p* = 7257; 0.475, *p* = 0.9977; 1.796, *p* = 3938} for {sleep control vs stress control; sleep control vs sleep-deprived; sleep control vs sleep rebound; stress control vs sleep-deprived; stress control vs sleep rebound; sleep-deprived vs sleep rebound}, respectively. ***D***, PDP (mean relative change of dF/F) from individual animals plotted against the animal's time of dissection. White circles represent control animals (allowed to sleep between ZT12 and ZT24). Pink circles represent sleep-deprived animals (on the shaker between ZT12 and ZT24).

Many of the most interesting questions about memory formation are ones that involve the interaction of plasticity with the animal's internal state. To test the ability of this preparation to retain traces of previous experience and allow interrogation of memory mechanisms at this level, we looked at the effect of sleep on subsequent *ex vivo* plasticity. Sleep is linked to memory across phyla. In *Drosophila*, perturbations of sleep have been shown to impair both memory formation (Bushey et al., 2007; Seugnet et al., 2008, 2009; X. Li et al., 2009; Donlea et al., 2014; Seidner et al., 2015) and consolidation (Ganguly-Fitzgerald et al., 2006; Donlea et al., 2009; Bushey et al., 2011; Dag et al., 2019; for review, see Goel et al., 2009; Diekelmann and Born, 2010; Dissel et al., 2015; Donlea, 2019). To determine whether the plasticity we observe in the MB is sleep-dependent, we mechanically sleep-deprived entrained (12:12 light:dark) flies for 12 h during the ZT12-ZT24 night period before using the AL+DA paradigm to induce the artificial memory at ZT0. Control flies were housed on a different shaker in the same incubator, but the shaker was turned off. A third group of flies received shaking for only the last 2 h of the night (ZT22-ZT24) to control for the acute physical stress that may result from the shaker. Figure 7*A* shows a schematic of our experimental design, and Figure 7*B* shows the minutes of sleep for each group during the relevant time windows. We found that sleep-deprived flies did not exhibit KC enhancement PDP, while both the sleep control group and the stress control group had normal PDP (Fig. 7*C*).

It is important to note that the stress control we used in this study is meant to control for acute stress, not the chronic stress that flies might experience when put on a shaker for 12 h. In many studies, the control for nighttime sleep deprivation via prolonged shaking is to sleep-deprive flies during the 12 h light period. We chose not to do this for several reasons. First, it would necessitate testing the flies for PDP/memory formation at ZT12 rather than ZT0, and STM has been shown to be influenced by the clock (Lyons and Roman, 2009; Flyer-Adams et al., 2020). Second, a recent study found that sleep deprivation during the light period causes significant sleep deprivation that is discharged as rebound sleep the next day (Wiggin et al., 2020); this would invalidate using daytime shaking as a control since it produces significant sleep debt. While shaking for the last 2 h of the night is not as stressful as 12 h of shaking, it is a better control for sleep deprivation since flies start waking up naturally in that time window, which means that there is very little sleep loss (as shown in Fig. 7*B*).

We then asked whether allowing sleep-deprived flies a period of recovery sleep could restore their ability to learn. As little as 2 h of rebound sleep was sufficient to restore the ability to induce the same level of α′3 PDP observed in the control groups (Fig. 7*C*). Interestingly, the 2 h minimum of rebound sleep that we found in our experiment to be required for rescuing the *ex vivo* plasticity is similar to the period reported *in vivo* to be required for sleep-deprived flies to restore the ability to form STM (X. Li et al., 2009). These findings demonstrate that our *ex vivo* paradigm not only forms the plasticity that is correlated with STM, but also accurately recapitulates the dynamics of the learning memory circuit and its interplay with the sleep circuit. These data also suggest that the effect of sleep deprivation on the memory circuit is downstream to sensory processing of both the CS and the US information and CS+US coincidence. This gradual recovery of the ability to form memory (Fig. 7*D*) may support previous indications that sleep deprivation likely impairs memory formation by downregulating dopamine receptors or other downstream molecules in the dopamine signaling pathway (Seugnet et al., 2008).

## Discussion

*Drosophila* neural circuits are traditionally studied by relating *in vivo* genetic and chemical manipulations with their consequent behavioral outcomes, from which circuit information can then be inferred (Olsen and Wilson, 2008; Simpson, 2009). More recently, the advent of *in vivo* calcium imaging allowed for tracing neural activity in actively behaving flies. Over more than a decade of such *in vivo* studies, the general circuit mechanisms of associative memory have been discovered, but there are limitations imposed by imaging the brain of an active intact fly (for review, see Adel and Griffith, 2021). These include the relatively low signal-to-noise ratio, the inaccessibility of multiple brain regions because of restrictions on imaging angles, the difficulty of doing acute pharmacological studies, and the possible confounds of studying the brain of a movement-restricted fly experiencing ongoing stress. Taking inspiration from the way the LTP hippocampal slice model revolutionized our understanding of mammalian memory (Bliss and Lomo, 1973), we provide here an *ex vivo* model of *Drosophila* memory which can overcome these limitations and offer a powerful preparation for studying *Drosophila* memory circuits. Importantly, this model provides a framework for investigating the dynamics of neural circuits in the fly brain.

Most of the previous studies investigating the associative learning circuit *ex vivo* have focused on mapping connectivity (Cohn et al., 2015; Barnstedt et al., 2016; Felsenberg et al., 2017, 2018; Zhao et al., 2018) or characterizing a specific biochemical pathway (Tomchik and Davis, 2009; Handler et al., 2019; Ueno et al., 2020). Only a few *ex vivo* studies (Wang et al., 2008; Ueno et al., 2013, 2017; Suzuki-Sawano et al., 2017) have focused on understanding MB circuit logic. In the LTE model developed by Ueno et al. (2013), pairing a stimulation of the CS and US pathways induced a potentiation of KC responses in the tips of the MB vertical lobes, but LTE did not fully recapitulate other characteristics of associative memory observed in intact flies. Here we develop a modified *ex vivo* model that resolves these discrepancies, showing that the paired activation of odor and punishment pathways induces appropriate plasticity at multiple nodes in the circuit: potentiation of KCs and suppression of MBONs. Several mechanisms for encoding those opposite forms of the plasticity have been proposed, including spike timing-dependent plasticity and activation of distinct dopaminergic receptors (for review, see Adel and Griffith, 2021). Spike timing-dependent plasticity mechanisms appear less likely as MBON suppression was shown to not require MBON spiking (Hige et al., 2015). Perhaps the strongest model so far comes from Handler et al. (2019) who showed that differences in the order of KCs activation and dopaminergic input activate distinct dopaminergic receptors, DopR1or DopR2, which encode MBON suppression or potentiation, respectively. It is important to note that both Hige et al. (2015) and Handler et al. (2019) studied the plasticity in MB medial lobes, while our study focused on MB vertical lobes, so our paradigm may be useful in gaining greater mechanistic insight into this sign transformation in the vertical lobes.

We show that PDP is localized to the MB α′3 compartment and not in α3, in alignment with most imaging studies in intact flies. Importantly, in this *ex vivo* preparation, punishment information is relayed to the MB through dopaminergic release from the PPL1 subset. Bath application of dopamine in our preparation does not interfere with the specificity of associative learning since PDP is exclusively formed in the cells that were active during the dopamine application. These data settle several inconsistencies between previous *ex vivo* studies (Ueno et al., 2013, 2017) and the majority of *in vivo* reports (Schwaerzel et al., 2003; Riemensperger et al., 2005; Schroll et al., 2006; Kim et al., 2007; Claridge-Chang et al., 2009; Mao and Davis, 2009; Aso et al., 2010, 2012, 2014b; Pech et al., 2013; Yamagata et al., 2016; Cognigni et al., 2018; Handler et al., 2019). We suggest that the genesis of the discrepancies was not because of any inherent difference between intact and *ex vivo* brains but was rather a consequence of technical considerations, including stimulation strength, dopamine concentration, and the sensor tools used (for a more complete discussion, see Adel and Griffith, 2021).

An *ex vivo* preparation that recapitulates the cardinal features of the circuits underlying associative memory formation should be useful for mechanistic studies at the molecular, cellular, and systems levels. We used our model to ask a new question about the innerworkings of the circuit at each of these levels. At the molecular level, we demonstrated the importance of normal levels of CaMKII by manipulating the 3′UTR of *CaMKII* mRNA. Deletion of this region of the *CaMKII* gene drastically reduces the amount of CaMKII protein in synaptic regions and blunts the ability to form STM and to generate a potentiation PDP in KC axons. Our data argue that the role of this molecule is downstream of the CS+US coincidence detector, as we observe a much weaker PDP in *CaMKII^Udel^* flies. Whether the behavioral defect is due solely to the KC PDP defect is not completely clear since CaMKII likely has active roles at other circuit nodes (Mitchell et al., 2021).

At the cellular level, we asked why STM and PDP form in the α′3 but not the nearby α3 compartment when both compartments respond to odors (Turner et al., 2008) and AL stimulation, and both receive dopaminergic input from the same PPL1 cluster (Masek et al., 2015). Previous work found that real odors cause activity in only 5%-12% of KCs and elicit a much higher spike rate in the α′β′ KCs than in the αβ KCs. We found that low-intensity AL stimulation (100 µAmps) elicits a stronger response in the α′3 than in the α3 compartment, while high-intensity AL-stimulation (200 µAmps) causes strong responses in the α3 compartment and recruits it to the learning circuit. Coupling this with our observation of lower dopamine release in α3 suggests a model in which odor presentation during associative learning causes subthreshold responses in αβ cells such that the CS+US coincidence detector is not triggered, while the stronger responses in the α′β′ cells bypass this threshold, allowing plasticity in the α′β′ cells only. This notion is in alignment with the previous finding that α′β′ cells have a lower firing threshold than αβ cells (Inada et al., 2017). Further, It is possible that long-term memory and the enhancement memory trace in the αβ KCs after repetitive space training (Yu et al., 2006) require a gradual potentiation of the αβ KC responses with every training session such that the responses bypass the coincidence detection threshold after several training sessions. Whether repetition of AL+DA pairings recruits PDP in the α3 compartment remains unclear. It is also yet to be determined whether shortcutting the circuit and recruiting αβ cells in the first training session reduces the need for multiple spaced training sessions in long-term memory formation.

In conclusion, we looked at the ability of the effects of prior experience, or brain state, on the memory circuit to be retained in the *ex vivo* preparation. Excitingly, we found that sleep-deprived flies could not form PDP, but that as little as 2 h of rest before dissection allowed the brain to recover PDP formation. The complete abolition of PDP in sleep-deprived flies at first and the gradual recovery in plasticity afterward (Fig. 7*D*) suggest that sleep converges on the memory circuit upstream of the CS+US coincidence detector. Whether this involves regulation of dopamine receptors in the MB during sleep remains to be determined. The ability to retain in some functional way the internal state of the brain will allow this preparation to be used to understand how memory formation is altered by global system alterations.
